# Effect of Omega-3 or Omega-6 Dietary Supplementation on Testicular Steroidogenesis, Adipokine Network, Cytokines, and Oxidative Stress in Adult Male Rats

**DOI:** 10.1155/2021/5570331

**Published:** 2021-06-28

**Authors:** Amira Moustafa

**Affiliations:** Department of Physiology, Faculty of Veterinary Medicine, Zagazig University, 44519 Zagazig, Egypt

## Abstract

This study was undertaken to elucidate the effect of omega-3 and omega-6 supplementation on the levels of different adipokines and cytokines, as well as the antioxidant system, in relation to male reproductive hormones and testicular functions. Adult male Sprague-Dawley rats were daily gavaged with either physiological saline (control group), sunflower oil (omega 6 group; 1 mL/kg body weight), or fish oil (omega-3 group; 1000 mg/kg body weight) for 12 weeks. The administration of omega-3 or omega-6 resulted in decreased serum concentrations of kisspeptin 1, gonadotropin-releasing hormone, luteinizing hormone, follicle-stimulating hormone, and testosterone. In addition, it downregulated the mRNA expression levels of steroidogenic genes. The intratesticular levels of apelin, adiponectin, and irisin were elevated while chemerin, leptin, resistin, vaspin, and visfatin were declined following the administration of either omega-3 or omega-6. The testicular concentration of interleukin 10 was increased while interleukin 1 beta, interleukin 6, tumor necrosis factor *α*, and nuclear factor kappa B were decreased after consumption of omega-3 or omega-6. In the testes, the levels of superoxide dismutase, catalase, glutathione peroxidase 1, and the total antioxidant capacity were improved. In conclusion, the administration of omega-3 or omega-6 adversely affects the process of steroidogenesis but improves the antioxidant and anti-inflammatory status of the reproductive system via modulating the levels of testicular adipokines.

## 1. Introduction

Based on their chemical nature, polyunsaturated fatty acids (PUFAs) are categorized into three groups: omega-3, omega-6, and omega-9. Linoleic acid, which is plentiful in vegetable oils such as sunflower oil, is the primary dietary source of omega-6 PUFA [1], and eicosapentaenoic acid (EPA) and docosahexaenoic acid (DHA) omega-3 PUFAs can be directly obtained from fish oils. Although diets rich in PUFAs are supposedly healthier, most people consume a higher amount of omega-6 PUFAs than required for normal physiological functioning, primarily as linoleic acid [2]. In particular, the Western diet is relatively low in omega-3 PUFAs and abundant in omega-6 PUFAs [3]. In both humans and animals, decreasing the consumption of omega-6 and increasing the intake of omega-3 are encouraged for better health [3]. Omega-3 fatty acids derived from fish oil have been shown to decrease inflammation, improve cardiac function [4], promote lipid degradation [5], and prevent neurological and psychiatric disorders [6]. Oxidative stress represents the imbalance between reactive oxygen species (ROS) production and the antioxidant defense mechanism. Spermatozoa are rich in mitochondria and are highly susceptible to ROS, and excess ROS production has been reported as a primary cause of male infertility [7]. Omega-3 PUFAs have been shown to enhance antioxidant enzyme activity and protect cells from excess ROS [8].

Adipokines work as autocrine, paracrine, and endocrine signaling molecules [9] that connect obesity and infertility. Adipokines are secreted primarily from adipose tissue, as well as lymphocytes, fibroblasts, and macrophages [10, 11]. Adipokines and their receptors are namely expressed in testicular cells: Sertoli cells, Leydig cells, and spermatozoa [12]. Moreover, several adipokines such as adiponectin, leptin, visfatin, chemerin, resistin, progranulin, and vaspin have been revealed in semen [13]. The expression of adipokines genes in the brain and pituitaries [14] suggests that adipokines could centrally act on the hypothalamus-pituitary axis and regulate reproductive functions.

In males, hypothalamic neurons secrete gonadotropin-releasing hormone (GnRH) that triggers the release of follicle-stimulating hormone (FSH) and luteinizing hormone (LH) from the pituitary gland, which in turn regulate testicular steroidogenesis and spermatogenesis [15]. In Leydig cells, testosterone is biosynthesized from cholesterol by a series of steroidogenic enzymes. First, the steroidogenic acute regulatory protein (StAR) mediates the process of importation of cholesterol into the mitochondria. In the mitochondria, cholesterol side-chain cleavage enzyme (CYP11A1) converts cholesterol into pregnenolone, which is then converted to progesterone by 3*β*-hydroxysteroid dehydrogenase (3*β*-HSD) [16]. 17*α*-hydroxylase/17, 20-lyase (CYP17A1), converts progesterone into androstenedione, which is converted to testosterone by 17*β*-hydroxysteroid dehydrogenase (17*β*-HSD), and testosterone is converted to estradiol by aromatase cytochrome P450 (CYP19) [16]. Thus, the synthesis of sex steroid hormones and the resultant male fertility could be influenced by the activity and/or expression of testicular steroidogenic enzymes.

The hormonal interaction between adipose tissue and the gonads is complex. Understanding the contribution of adipokines in testicular functions is of special interest, and little is known about the effect of omega-3 and omega-6 supplementation on testicular adipokines, particularly their potential effects on spermatogenesis and steroidogenesis. Therefore, the present study is aimed at assessing adipokines (leptin, adiponectin, chemerin, apelin, visfatin, vaspin, resistin, and irisin) in testicular tissues, examining the mRNA expression of steroidogenic genes, and determining the anti-inflammatory and antioxidant potentials of omega-3 and omega-6.

## 2. Materials and Methods

### 2.1. Tested Compounds

Omega-3 plus capsules were purchased from the SEDICO Company (Cairo, Egypt) and were 1000 mg fish oil soft gel capsules containing 13% EPA and 9% DHA.

Sunflower oil was purchased from Arma Oils Co. (10th of Ramadan City, Egypt) and contained a higher concentration of linoleic acid (polyunsaturated omega-6; 59%) than oleic acid (monounsaturated omega-9; 30%) [17].

### 2.2. Animal Experiment

Thirty adult Sprague-Dawley male rats (body weight, 300–330 g) were purchased from the animal research center (Faculty of Veterinary Medicine, Zagazig University). Rats were housed with two rats per cage under a light-dark cycle of 12 : 12 h in a controlled room with a constant temperature of 22°C and 50% humidity. Rats were acclimatized to the housing conditions for two weeks prior to the experiment, and pellet food and water were supplied ad libitum. Rats were randomly assigned to the control group (*n* = 10), the sunflower oil group (*n* = 10), or the omega-3 group (*n* = 10). For 12 weeks, rats were administered the following by oral gavage each day (between 8:00 and 9:00 a.m.): (1) physiological saline (1 mL/kg body weight/d; the control group); (2) sunflower oil, rich in omega-6 PUFAs [1] (1 mL/kg body weight/d; the sunflower group); and (3) omega-3 capsules, rich in omega-3 PUFAs [18] (1000 mg/kg body weight/d; the omega-3 group). Bodyweight and food intake were measured weekly. The amount of oils and saline were readjusted with the increase in body weight.

The study was approved by the Institutional Animal Care and Use Committee of the Faculty of Veterinary Medicine, Zagazig University (Permit Number: ZU-IACUC/2/F/103/2020).

### 2.3. Sample Collection

Rats were sacrificed by rapid decapitation [19] after overnight fasting, and trunk blood was collected and centrifuged at 4000 rpm for 15 minutes at 4°C. Then, the serum was separated and stored at –20°C until analysis. The testicular homogenates for cytokines, adipokines, and hormone measurements were prepared by suspending the testes in ice-cold phosphate buffer (0.1 M, 7.4), disrupting the tissues by a homogenizer, and centrifugation at 3000 rpm for 20 min. The supernatants were aliquoted and used for different measurements. For histological and immunohistochemical examinations, the testes, epididymides, prostate glands, and seminal vesicles were excised, weighed, and fixed in 10% neutral buffered formalin solution. For histological examination, the testicular sections were stained with hematoxylin and eosin (H&E). Part of the testes was rapidly excised, weighed, snap-frozen in liquid nitrogen, and stored at −80°C for gene expression.

### 2.4. Analysis of Semen Quality

The cauda epididymides were immediately excised after sacrifice. The tissues were sliced with a scalpel several times in a Petri dish containing 2 mL normal saline at 37°C to release spermatozoa, and the percentage of sperm motility was assessed using a light microscope (×40) as described previously [20–22]. Semen samples were diluted five times (*v*/*v*) with normal saline containing a few drops of formalin (40%) to kill the spermatozoa, and sperm was counted using a hemocytometer [23]. Morphological abnormalities of the sperm were identified, and the results were expressed as percentages [24].

### 2.5. Measurement of Hormones

The enzyme-linked immunosorbent assay (ELISA; Cusabio Biotech Co., Wuhan, China) was used to determine the concentrations of kisspeptin 1 (CSB-E13434r), FSH (CSB-E06869r), LH (CSB-E12654r), and testosterone (CSB-E05100r) according to the manufacturer's protocol. The analytical sensitivities of these assays for kisspeptin 1, FSH, LH, and testosterone were 0.039 ng/mL, 0.07 mIU/mL, 0.15 mlU/mL, and 0.06 ng/mL, respectively. The intra-assay and interassay coefficients of variation were below 10%.

GnRH, prolactin, and 17*β* estradiol (E2) concentrations were estimated using an ELISA kit (MyBioSource Inc., San Diego, CA, USA; Cat No. MBS268023, MBS2512489, and MBS263466, respectively) according to the manufacturer's instructions. The analytical sensitivities of this assay for GnRH, prolactin, and E2 were 5.0 pg/mL, 0.469 ng/mL, and 5.0 pg/mL, respectively. The coefficients of variation were less than 8%. The absorbance was measured at 450 nm using a *DNM-9602* microplate reader (PERLONG, Beijing, China).

The testicular concentration of prostaglandin F2 alpha (PGF2*α*) was estimated using a rat ELISA kit from MyBioSource Inc. (San Diego, CA, USA; Cat No. MBS764597), and prostaglandin E2 (PGE2) was assessed by ELISA kit from LifeSpan BioScience, Inc. (WA, USA; Cat No. LS-F27545) according to the manufacturer's protocol at an absorbance of 450 nm. The analytical sensitivities of these assays for PGF2*α* and PGE2 were <4.688 pg/mL and 0.1 ng/mL, respectively. The intra- and interassay coefficients of variation were <8% and <10%, respectively.

### 2.6. Measurement of Testicular Adipokines and Cytokines

Rat ELISA kits (Cusabio Biotech Co., Wuhan, China) were used to measure the concentrations of testicular adipokines, namely adiponectin (Cat No. CSB-E07271r), apelin (Cat No. CSB-E13435r), leptin (Cat No. CSB-E07433r), resistin (Cat No. CSB-E06885r), vaspin (Cat No. CSB-E09813r), and visfatin (Cat No. CSB-E08941r), following the manufacturer's instructions (analytic sensitivities: 0.039 ng/mL, 0.078 ng/mL, 0.068 ng/mL, 31.25 pg/mL, 7.8 pg/mL, and 0.78 ng/mL, respectively). The intra-assay and interassay precisions were <8% and <10%, respectively.

Chemerin concentrations were estimated using a rat ELISA kit (MyBioSource Inc., San Diego, CA, USA; Cat No. MBS760975), and testicular irisin levels were measured using another ELISA kit (Wuhan Fine Biotech Co. Ltd., China; Cat No. ER1486) according to the manufacturer's protocol (analytical sensitivities, 18.75 pg/mL and 0.469 ng/mL, respectively). A microplate reader (DNM-9602, PERLONG, Beijing, China) was used to measure the absorbance at 450 nm.

The level of the proinflammatory cytokine interleukin 1 beta (IL-1B) was assessed using an ELISA kit (MyBioSource Inc., San Diego, CA, USA; Cat No. MBS825017) with a sensitivity of 15 pg/mL. Rat ELISA kits (Cusabio Biotech Co., Wuhan, China) were used to measure the intratesticular concentrations of interleukin 6 (IL-6; Cat No. CSB-E04640r), interleukin-10 (IL-10; Cat No. CSB-E04595r), tumor necrosis factor-*α* (TNF-*α*; Cat No. CSB-E11987r), and nuclear factor kappa B (NF-*κ*B; Cat No. CSB-E13148r) according to the manufacturer's instructions (analytical sensitivities, 0.078 pg/mL, 0.78 pg/mL, 1.56 pg/mL, and 0.39 pg/mL, respectively). The intra-assay and interassay precisions were below 8% and 10%, respectively.

### 2.7. Serum Lipid Analysis

Serum concentrations of free fatty acids were assessed by a rat ELISA kit (Cusabio Biotech Co., Wuhan, China; Cat No. CSB-E08770r) with a sensitivity of 4 ng/mL. Serum triglycerides, cholesterol, HDL-cholesterol, LDL-cholesterol, and VLDL-cholesterol concentrations were determined using the Reactivos Spinreact colorimetric assay kits (Barcelona, Spain) according to the manufacturer's instructions.

### 2.8. Spermatozoa Lipid Analysis

Spermatozoa were separated by centrifuging semen at 1000 g for 15 min. seminal plasma was carefully removed, filtered, and stored at −80°C until use. The remaining pellet was washed three times with phosphate-buffered saline (PBS), then resuspended in 0.5 mL PBS, homogenized, and sonicated. The concentrations of total lipids, triglycerides, and cholesterol were assessed using the Reactivos Spinreact colorimetric assay kits (Barcelona, Spain) following the manufacturer's instructions. Phospholipids were measured calorimetrically at 570 nm using an ELISA kit (Abnova, Taipei, Taiwan; Cat No. KA1635) according to the manufacturer's protocol. The levels of arachidonic acid were estimated using a quantitative ELISA kit (Creative Diagnostics, New York, USA; Cat No. DEIA-Bj2354) at 450 nm according to the manufacturer's instructions.

### 2.9. Analysis of Fructose, *α*-Glucosidase, and Acid Phosphatase in Seminal Plasma

Fructose concentrations in the seminal plasma were measured spectrophotometrically using an assay kit (Sigma Chemical Company, St. Louis, MO, USA), and the absorbance values were read at 340 nm wavelength. Alpha-glucosidase was estimated using a rat ELISA kit (Cusabio Biotech Co., Wuhan, China; Cat No. CSB-E09906r), and the concentrations of acid phosphatase were determined using another ELISA kit (Reagent Genie, Dublin, Ireland) following the manufacturer's protocol. The intra-assay and interassay precisions were below 8% and 10%, respectively.

### 2.10. Immunohistochemistry of the Androgen Receptor

The specimens of testes, seminal vesicles, prostate glands, and epididymides were fixed in 10% neutral buffered formalin solution, and the paraffin sections were prepared [25]. An UltraVision LP large volume detection system (Thermo Fisher Scientific, Fremont, USA; Cat No. TP-060-HL) was used for the detection of immunohistochemical expression and the localization of androgen receptor in the target tissues. The tissue slices (4 *μ*m thick) were deparaffinized and rehydrated, and endogenous peroxidase activity was blocked using hydrogen peroxide (H_2_O_2_; 3% solution in methanol) for 10 min. Antigen retrieval was performed by heating the tissue sections in 10 mM citrate buffer (pH, 6.0) for 5 min, followed by cooling at room temperature for 20 min. After rinsing three times in PBS, the nonspecific background staining was blocked by incubating the tissue slices with Ultra V Block for 5 minutes at room temperature. Then, the tissue slices were incubated overnight at 4°C with a rabbit polyclonal antibody against androgen receptor (200 *μ*g/mL; Thermo Fisher Scientific, Fermont, USA; Cat No. RB-9030-R7). The slices were rinsed in PBS, incubated with a biotinylated goat anti-polyvalent for 10 minutes at room temperature, followed by incubation with streptavidin peroxidase for another 10 minutes at room temperature. 3,3′-Diaminobenzidine (DAB; Quartett Immunodiagnostika GmbH, Berlin, Germany) was used as a chromogen, and sections were counterstained with hematoxylin. Negative control slides were incubated without the primary antibody. The mean intensity of the brown staining was obtained from five random fields in each slide using the ImageJ Fiji software version 1.53f. The formula *FI* = 255 − *i* was used to calculate the final DAB intensity, where *FI* is the final DAB intensity, 255 is the maximum intensity for 8-bit images, and *i* is the mean DAB intensity obtained from the software [26].

### 2.11. RNA Extraction and cDNA Synthesis

Total RNA was extracted from the testes of all groups using TRIzol (Invitrogen; Thermo Fisher Scientific, Inc.) according to the manufacturer's instructions as previously indicated [20, 22]. The NanoDrop ND-1000 spectrophotometer (NanoDrop Technologies, Wilmington, Delaware, USA) was used to determine the concentration and purity of the total RNA. A HiSenScript™ RH (-) cDNA synthesis kit (iNtRON Biotechnology Co., South Korea) was utilized to reverse-transcribe the total RNA into cDNA following the manufacturer's protocol. Then, the reaction mixtures were incubated in a Veriti 96-well thermal cycler (Applied Biosystems, Foster City, CA) for 60 minutes at 45°C, followed by 10 minutes at 85°C.

### 2.12. Real-Time Polymerase Chain Reaction

A Stratagene Mx3005P system (Agilent Technologies, Santa Clara, CA, USA) and RbTaq™ qPCR 2X premix (SYBR green with low ROX; Enzynomics, Daejeon, Korea) were used to perform real-time reverse transcription-polymerase chain reaction (RT-PCR) following the manufacturer's instructions. The expression levels of the target mRNAs were quantified relative to the level of the GAPDH (housekeeping gene). The oligonucleotide primer sequences [27] (Eurofins Genomics, Ebersberg, Germany) are listed in Table 1.

### 2.13. Measurement of the Testicular Levels of the Antioxidant Enzymes and Lipid Peroxidation

The testicular levels of catalase (CAT), glutathione peroxidase 1 (GPx1), and ROS were determined using rat ELISA kits (MyBioSource Inc., San Diego, CA, USA; Cat No. MBS2600683, MBS451149, and MBS164653, respectively) according to the manufacturer protocol (sensitivities: 0.06 ng/mL, 0.63 ng/mL, and 2.49 U/mL, respectively). The intra-assay and interassay coefficients of variation were <8% and 10%, respectively. The levels of hydrogen peroxide (H_2_O_2_) and total antioxidant capacity (TAC) were measured using ELISA kits (Cell Biolabs, Inc., OxiSelect*™*, San Diego, CA, USA; Cat No. STA-844 and STA-360, respectively). The absorbance was measured at 540 nm and 490 nm, respectively, using a DNM-9602 microplate reader (PERLONG, Beijing, China). The concentrations of superoxide dismutase (SOD) were estimated using rat ELISA kits according to the manufacturer's instructions (Cusabio Biotech Co., Wuhan, China; Cat No. CSB-E08555r) with a sensitivity of 1.95 U/mL. The lipid peroxidation marker malondialdehyde (MDA) was determined using a colorimetric assay kit (Elabscience, Inc., Texas, USA; Cat No.E-BC-K025-S) with an analytic sensitivity of 0.38 nmol/mL according to the manufacturer's instructions.

### 2.14. Data Analysis

Data are illustrated as the means ± the standard error of the means and were analyzed using one-way analysis of variance. Post hoc multiple comparisons were performed using Tukey's test. The statistical significance was set at *P* < 0.05.

## 3. Results

### 3.1. Effect of Omega-3 and Sunflower Oil on Body Weight and Sperm Parameters

**Figure 1:**
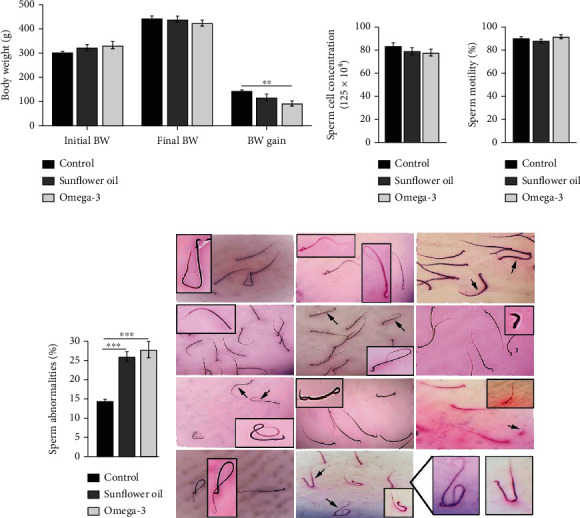
Changes in bodyweight and sperm parameters. (a) Changes in initial and final body weight and body weight gain (g) in the control, sunflower oil, and omega-3 groups. (b–d) Sperm count, motility (%), and abnormalities (%), respectively, in the control, sunflower oil, and omega-3 groups. Data are mean ± SEM (*n* = 10/group). ^∗∗^*P* < 0.01 and ^∗∗∗^*P* < 0.001 by Tukey's multiple comparison post hoc test. (e) Photomicrographs of sperm abnormalities including: tail abnormalities, bent neck, detached head and tail, and amorphous head.

The omega-3 group exhibited significantly less body weight gain than the other two groups (Figure 1(a); *P* < 0.01). Neither omega-3 nor sunflower oil administration affected the sperm cell concentration or sperm motility (Figures 1(b) and 1(c), respectively), but both significantly increased the number of morphologically abnormal sperms (Figure 1(d); *P* < 0.01). The observed sperm abnormalities included tail abnormalities (looped tail, curved tail, coiled tail, and detached tail), head abnormalities (amorphous head and detached head), and bent neck (Figure 1(e)).

### 3.2. Effect of Omega-3 and Sunflower Oil on Serum and Sperm Lipid Profile

**Figure 2:**
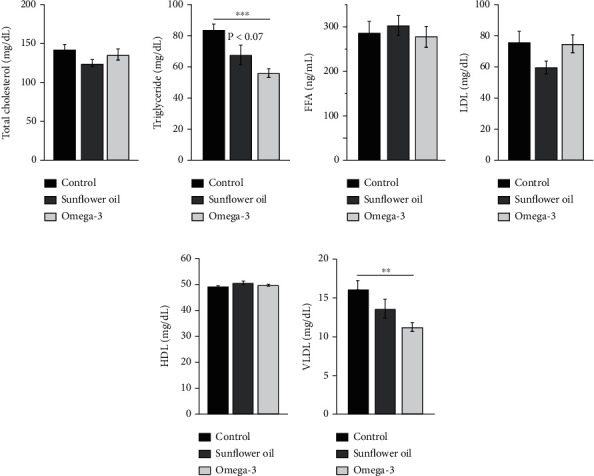
Changes in serum lipid profile. (a) Total cholesterol (mg/dL), (b) triglyceride (mg/dL), (c) free fatty acids (FFA, ng/mL), (d) low-density lipoprotein (LDL, mg/dL), (e) high-density lipoprotein (HDL, mg/dL), and (F) very low-density lipoprotein (VLDL, mg/dL) in the control, sunflower oil, and omega-3 groups. Data are expressed as mean ± SEM (*n* = 10/group). ^∗∗^*P* < 0.01 and ^∗∗∗^*P* < 0.001 by Tukey's multiple comparison post hoc test.

Serum levels of total cholesterol, free fatty acids, LDL, and HDL revealed no significant changes among different groups (Figures 2(a), 2(c), 2(d), and 2(e)). However, the levels of triglycerides and VLDL were significantly declined following omega-3 administration (Figures 2(b) and 2(f); *P* < 0.001 and *P* < 0.01, respectively). In the sunflower oil group, triglycerides and VLDL concentrations tended to decrease, but the results remained insignificant (Figures 2(b) and 2(f)).

**Figure 3:**
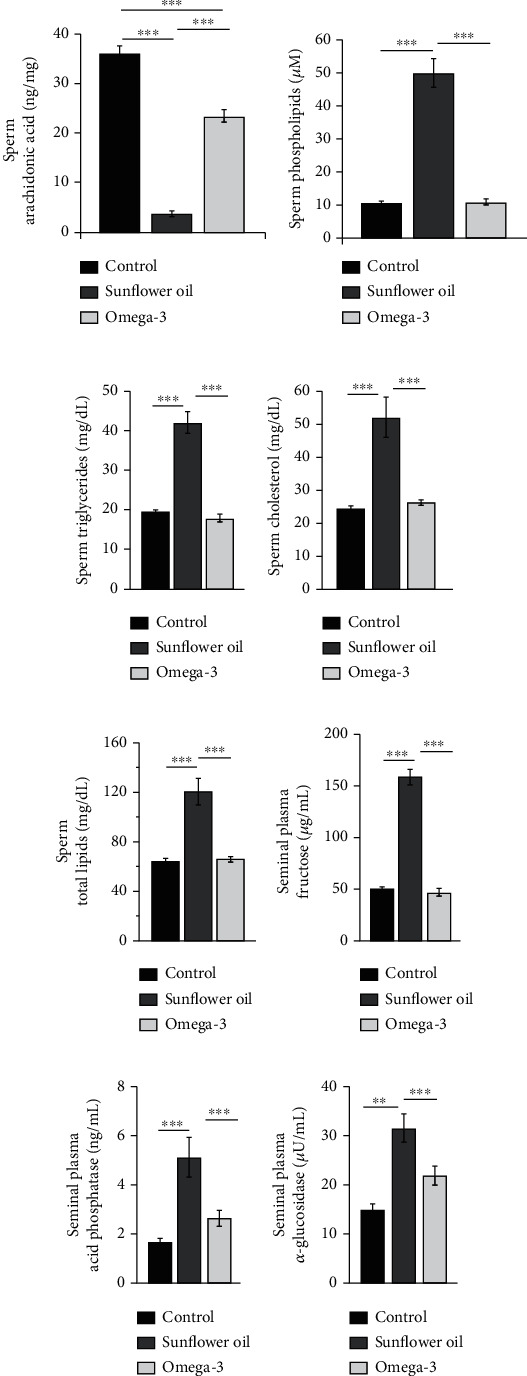
Changes in spermatozoa lipid composition and seminal plasma biochemistry. Spermatozoa lipid contents, namely, (a) arachidonic acid (ng/mg), (b) phospholipids (*μ*M), (c) triglycerides (mg/dL), (d) cholesterol (mg/dL), and (e) total lipids (mg/dL) in the control, sunflower oil, and omega-3 groups. Seminal plasma levels of (f) fructose (*μ*g/mL), (g) acid phosphatase (ng/mL), and (h) *α*-glucosidase (*μ*U/mL) in the control, sunflower oil, and omega-3 groups. Data are presented as mean ± SEM (*n* = 10/group). ^∗∗^*P* < 0.01 and ^∗∗∗^*P* < 0.001 by Tukey's multiple comparison post hoc test.

Sperm concentrations of arachidonic acid were significantly decreased in both omega-3 and sunflower oil groups compared with the control group (Figure 3(a); *P* < 0.001) and sharply reduced in the sunflower oil group compared with the omega-3 group (Figure 3(a); *P* < 0.001). Sperm levels of phospholipids, triglycerides, cholesterol, and total lipids were significantly elevated following sunflower oil administration compared with the control and omega-3 groups (Figures 3(b)–3(e); *P* < 0.001).

### 3.3. Effect of Omega-3 and Sunflower Oil on the Levels of Seminal Plasma Fructose, *α*-Glucosidase, and Acid Phosphatase

The levels of fructose (seminal vesicles), *α*-glucosidase (epididymides), and acid phosphatase (prostate glands) in seminal plasma were significantly increased in the sunflower oil group compared with the control and omega-3 groups (Figures 3(f)–3(h); *P* < 0.001).

### 3.4. Effect of Omega-3 and Sunflower Oil on Serum and Testicular Levels of Reproductive Hormones

**Figure 4:**
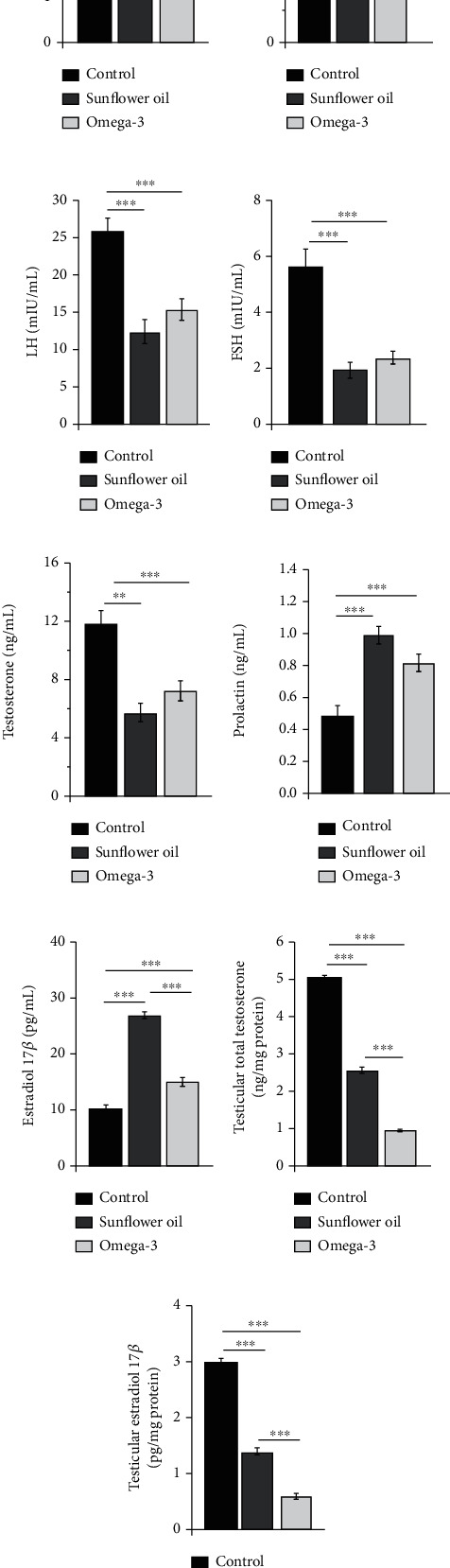
Changes in serum and testicular hormones concentrations. Serum concentrations of (a) kisspeptin (ng/mL), (b) gonadotropin-releasing hormone (GnRH; pg/mL), (c) luteinizing hormone (LH; mIU/mL), (d) follicle-stimulating hormone (FSH; mIU/mL), (e) testosterone (ng/mL), (f) Prolactin (ng/mL), and (g) estradiol 17*β* (E2; pg/mL) in the control, sunflower oil, and omega-3 groups. Testicular levels of (h) total testosterone (ng/mg) and (i) estradiol 17*β* (E2; pg/mg) in the control, sunflower oil, and omega-3 groups. Data are shown as mean ± SEM (*n* = 10/group). ^∗∗^*P* < 0.01 and ^∗∗∗^*P* < 0.001 by Tukey's multiple comparison post hoc test.

Serum concentrations of kisspeptin, GnRH, LH, FSH, and testosterone were significantly reduced in the omega-3 and sunflower oil groups compared with the control group (Figures 4(a)–4(e); *P* < 0.001 and *P* < 0.01, respectively). The levels of prolactin and E2 were augmented in the omega-3 or sunflower oil groups compared with the control group (Figures 4(f) and 4(g); *P* < 0.001). However, the sunflower oil group showed a much higher E2 than the omega-3 group (Figure 4(g); *P* < 0.001).

The intratesticular concentrations of testosterone were significantly reduced in the sunflower oil and omega-3 group compared with the control group (Figure 4(h); *P* < 0.001), and such inhibition was significantly higher in the omega-3 group than in the sunflower oil group (Figure 4(h); *P* < 0.001). Moreover, the intratesticular levels of E2 were significantly reduced in the sunflower oil and omega-3 groups compared with the control group (Figure 4(i); *P* < 0.001), and the reduction was higher in the omega-3 group than in the sunflower oil group (Figure 4(i); *P* < 0.001).

### 3.5. Effect of Omega-3 and Sunflower Oil on the Steroidogenesis Genes Expression

**Figure 5:**
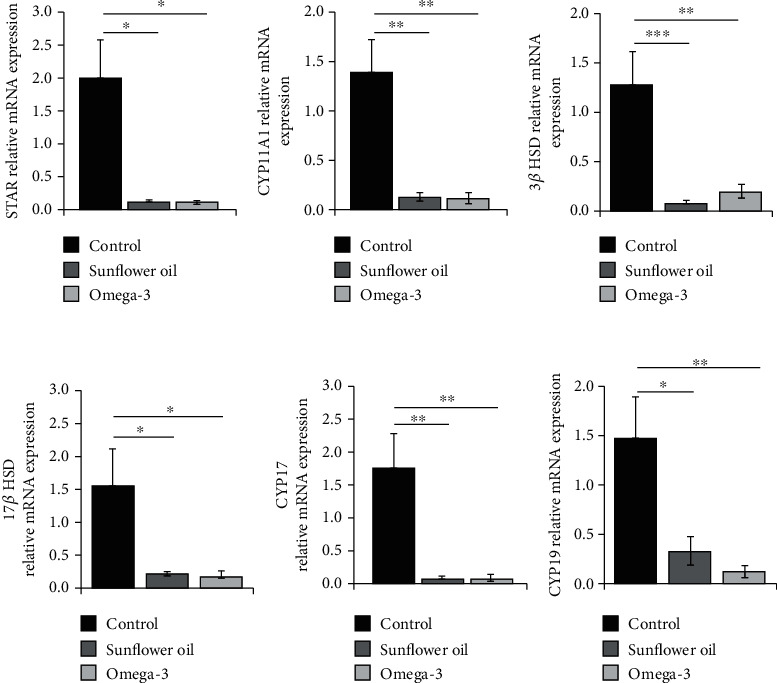
Changes in the expression levels of testicular steroidogenic genes. Relative expression levels of (a) Steroidogenic acute regulatory protein (*StAR*), (b) cholesterol side-chain cleavage enzyme (*CYP11A1*), (c) 3*β*-hydroxysteroid dehydrogenase (*3β-HSD*), (d) 17*β*-hydroxysteroid dehydrogenase (*17β-HSD*), (e) 17*α*-hydroxylase/17, 20-lyase (*CYP17A1*), and (f) cytochrome P450 aromatase (*CYP19*) in the control, sunflower oil, and omega-3 groups. Data are expressed as mean ± SEM. ^∗^*P* < 0.05, ^∗∗^*P* < 0.01, and ^∗∗∗^*P* < 0.001 by Tukey's multiple comparison post hoc test.

To investigate the mechanism responsible for the inhibitory effect of sunflower oil and omega-3 on the process of steroidogenesis, RT-PCR was performed to detect the changes in genes regulating steroidogenesis. *STAR* mRNA levels were significantly reduced in the sunflower oil and omega-3 groups (Figure 5(a); *P* < 0.05). In addition, the expression levels of the *CYP11A1* and *3β-HSD* genes were downregulated in the sunflower oil and omega-3 groups (Figures 5(b) and 5(c); *P* < 0.01 and *P* < 0.001). The expression levels of *17β-HSD* mRNA were significantly decreased in both sunflower oil and omega-3 groups (Figure 5(d); *P* < 0.05). The administration of sunflower oil or omega-3 significantly downregulated the mRNA expression levels of *CYP17A1* (Figure 5(e); *P* < 0.01). The expression levels of the *CYP19* gene were significantly decreased by the sunflower oil and omega-3 administration (Figure 5(f); *P* < 0.05 and *P* < 0.01, respectively).

### 3.6. Effect of Omega-3 and Sunflower Oil on the Immunohistochemical Expression of the Androgen Receptor

**Figure 6:**
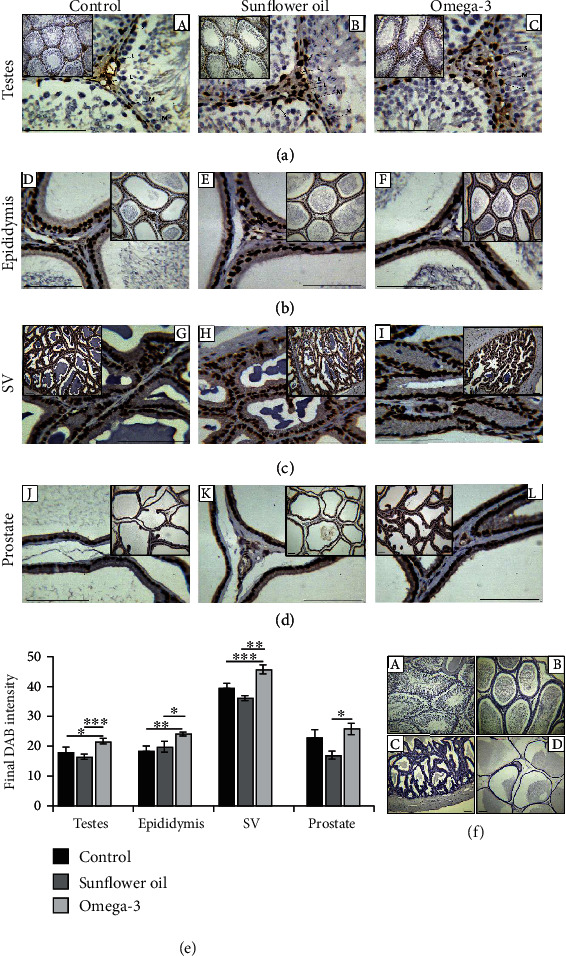
Immunohistochemical staining of androgen receptor in different reproductive tissues. Immunohistochemical distribution of androgen receptor in (a) testes, (b) epididymis, (c) seminal vesicle (SV), and (d) prostate of the control (A, D, G, and J, respectively), sunflower oil (B, E, H, and K, respectively), and omega-3 (C, F, I, and L, respectively) groups. (e) Quantitative analysis of the nuclear immunostaining of androgen receptor in the control, sunflower oil and omega-3 groups. Data are calculated by the ImageJ *Fiji* software and presented as mean ± SEM. ^∗^*P* < 0.05, ^∗∗^*P* < 0.01, and ^∗∗∗^*P* < 0.001 by Tukey's multiple comparison post hoc test. (f) Negative tissue sections of (A) testes, (B) epididymis, (C) seminal vesicle, and (D) prostate. Scale bar = 100 *μ*m.

In testicular tissues (Figure 6(a)), androgen receptor expression was revealed in the nuclei of spindle-shaped Leydig cells, myoid cells, and Sertoli cells in all examined groups. However, this expression was significantly increased in the omega-3 group compared with the control group (Figure 6(e); *P* < 0.05). No significant difference was noticed between the control group and the sunflower oil group, but the intensity of immunostaining was reduced in the sunflower oil group compared with the omega-3 group (Figure 6(e); *P* < 0.001). Sertoli cells were identified by their large columnar or pyramidal shape and oval nucleus and are attached to the basal lamina of the basement membrane. Leydig cells are characterized by a polyhedral shape with large prominent ovoid nuclei and are present in the connective tissues among the seminiferous tubules. Myoid cells are smooth muscle cells that surround the seminiferous tubules and are characterized by elongated shapes with spindle-shaped nuclei.

The epididymides of all three groups showed androgen receptor staining in the nuclei of interstitial stromal cells, and epithelial cells with robust staining of the latter (Figure 6(b)). The intensity of the androgen receptor staining was augmented in the omega-3 group compared with the control and sunflower oil groups (Figure 6(e); *P* < 0.01 and *P* < 0.05, respectively). The immunohistochemical staining of the androgen receptor in the seminal vesicles was located in the nuclei of the epithelial cells and fibromuscular stroma of all three groups (Figure 6(c)). The nuclear staining intensity was increased in the omega-3 group compared with the control and sunflower oil groups (Figure 6(e); *P* < 0.001 and *P* < 0.01, respectively). In the prostate gland, androgen receptor immunoreactivity was localized to the nuclei of the epithelial cells in all three groups (Figure 6(d)). However, the nuclear staining intensity was significantly decreased in the sunflower oil group compared with the omega-3 group (Figure 6(e); *P* < 0.05). The immunoreactivity of the androgen receptor was undetectable in the control sections of testes, epididymis, seminal vesicle, and prostate, which were incubated without the primary antibody (Figure 6(f)).

### 3.7. Effect of Omega-3 and Sunflower Oil on the Testicular Levels of Cytokines and Prostaglandins

**Figure 7:**
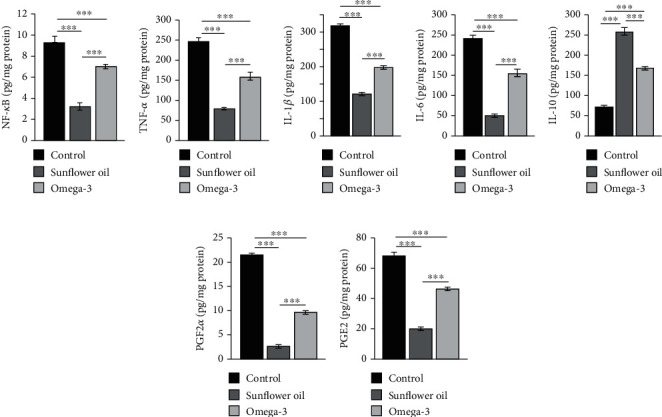
Changes in the testicular levels of cytokines and prostaglandins. Testicular concentrations of (a) NF-*κ*B (pg/mg), (b) tumor necrosis factor alpha (TNF-*α*; pg/mg), (c) interleukin-1*β* (IL-1*β*; pg/mg), (d) interleukin-6 (IL-6; pg/mg), (e) interleukin-10 (IL-10; pg/mg), (f) prostaglandin F2 alpha (PGF2*α*; pg/mg), and (g) prostaglandin E2 (PGE2; pg/mg) in the control, sunflower oil, and omega-3 groups. Data are shown as mean ± SEM (*n* = 10/group). ^∗∗∗^*P* < 0.001 by Tukey's multiple comparison post hoc test.

The testicular levels of NF-*κ*B, TNF-*α*, IL-1*β*, and IL-6 were significantly attenuated in the sunflower oil and omega-3 groups compared with the control group (Figures 7(a)–7(d); *P* < 0.001). However, the attenuation was considerably greater in the sunflower oil group than in the omega-3 group (Figures 7(a)–7(d); *P* < 0.001). Compared with the control group, the sunflower oil and omega-3 groups showed a significant increase in the testicular levels of IL-10 (Figure 7(e); *P* < 0.001), with more prominent changes in the omega-3 group than in the sunflower oil group. In the testes, the levels of both PGF2*α* and PGE2 were significantly diminished in the sunflower oil and omega-3 groups compared with the control group (Figures 7(f) and 7(g); *P* < 0.001), and the reduction was significantly larger in the sunflower oil group than in the omega-3 group (Figures 7(f) and 7(g); *P* < 0.001).

### 3.8. Effect of Omega-3 and Sunflower Oil on the Testicular Concentrations of Adipokines

**Figure 8:**
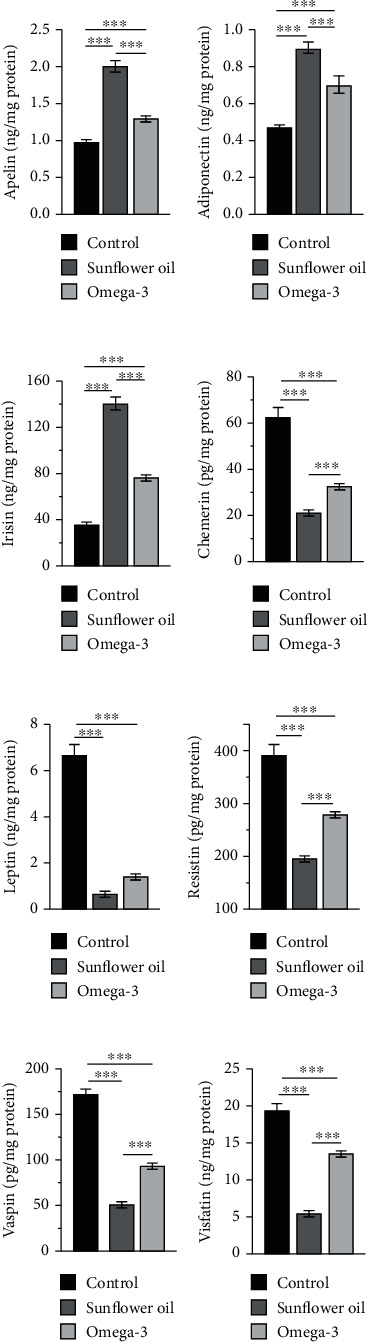
Changes in the testicular levels of different adipokines. Testicular levels of (a) apelin (ng/mg), (b) adiponectin (ng/mg), (c) irisin (ng/mg), (d) chemerin (pg/mg), (e) leptin (ng/mg), (f) resistin (pg/mg), (g) vaspin (pg/mg), and (h) visfatin (ng/mg) in the control, sunflower oil, and omega-3 groups. Data are expressed as mean ± SEM (*n* = 10/group). ^∗∗∗^*P* < 0.001 by Tukey's multiple comparison post hoc test.

The testicular levels of adipokines, namely apelin, adiponectin, and irisin, were significantly increased in the sunflower oil and omega-3 groups compared with the control group, and the increment was larger in the sunflower oil group than in the omega-3 group (Figures 8(a)–8(c); *P* < 0.001). However, compared with the control group, the sunflower oil and omega-3 groups had significantly decreased testicular levels of chemerin, leptin, resistin, vaspin, and visfatin, and the reduction was larger in the sunflower oil group than in the omega-3 group (Figures 8(d)–8(h); *P* < 0.001).

### 3.9. Effect of Omega-3 and Sunflower Oil on the Testicular Levels of the Antioxidant Enzymes, ROS, and TAC

**Figure 9:**
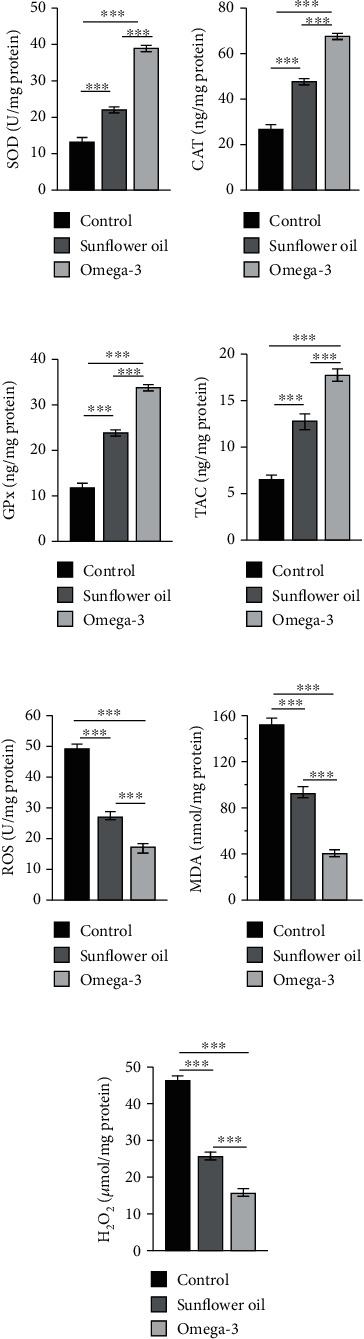
Testicular tissue levels of antioxidant enzymes, total antioxidant capacity, and reactive oxygen species. Testicular tissue levels of (a) superoxide dismutase (SOD; U/mg), (b) catalase (ng/mg), (c) glutathione peroxidase (GPx; ng/mg), (d) total antioxidant capacity (TAC; ng/mg), (e) reactive oxygen species (ROS; U/mg), (f) malondialdehyde (MDA; nmol/mg), and (g) hydrogen peroxide (H_2_O_2_; *μ*mol/mg) in the control, sunflower oil, and omega-3 groups. Data are expressed as mean ± SEM (*n* = 10/group). ^∗∗∗^*P* < 0.001 by Tukey's multiple comparison post hoc test.

The testicular levels of SOD, CAT, GPx, and TAC were significantly elevated in the sunflower oil and omega-3 groups compared with the control group (Figures 9(a)–9(d); *P* < 0.001), with more elevation in the omega-3 group than in the sunflower oil group (Figures 9(a)–9(d); *P* < 0.001). Conversely, the levels of ROS, MDA, and H_2_O_2_ were lower in both sunflower oil and omega-3 groups than in the control group (Figures 9(e)–9(g); *P* < 0.001), with a greater reduction in the omega-3 group than in the sunflower oil group (Figures 9(e)–9(g); *P* < 0.001).

## 4. Discussion

Phospholipids and cholesterol are significant elements of human spermatozoa plasma membranes and are necessary for the fluidity, permeability, and capacitation of the membrane [28], and alteration in spermatozoa lipid contents has been linked to male infertility [29]. The results of the present study showed that only sunflower oil administration increases the spermatozoa content of phospholipids, triglycerides, cholesterol, and total lipids, which may be attributed to sunflower oil being rich in linoleic acid. However, the spermatozoa content of arachidonic acid was decreased after the administration of omega-3 or sunflower oil. Increasing the intake of linoleic acid has been demonstrated to increase [30] or decrease the tissue content of arachidonic acid [31, 32] where increased linoleic acid in the diet may compete with arachidonic acid for deacylation into the phospholipids [31–33]. The ingestion of omega-3 PUFAs from fish oil has been shown to reduce the membrane levels of arachidonic acid and decrease concomitantly the potential arachidonic acid synthesis of eicosanoids [34, 35]. The metabolism of arachidonic acid by cyclooxygenases results in the synthesis of 2-series prostaglandins: PGE2, PGI2, PGD2, and PGF2*α*. In the present study, the administration of omega-3 or sunflower oil for 12 weeks significantly reduced the circulating levels of PGE2 and PGF2*α*. Fish oil and omega-3 PUFA have been shown to diminish PGE2 production [36, 37], and the administration of 6 g/d DHA abolished endotoxin-stimulated mononuclear cell production of PGE2 [38].

Seminal plasma contains a mixture of various parameters originating from the epididymis and accessory sex glands, which are essential for sperm maturation and fertilization capacity [39]. Fructose is the primary energy source for sperm activity, and a decline in sperm motility and fertilization capability has been associated with low seminal fructose levels [40]. Acid phosphatase has been reported to be linked to semen concentration [41] and the carbohydrate metabolism of spermatozoa [42]. Furthermore, the activity of the seminal plasma *α*-glucosidase indicates the functional condition of the epididymis [43] and is closely correlated with ejaculate volume, sperm concentration, and acrosome reaction [44]. Decreased levels of fructose and low activity of *α*-glucosidase have been associated with high levels of omega-3 or omega-6 fatty acids in boar seminal plasma [45]. In the present study, although sunflower oil administration induced increases in the levels of fructose, acid phosphatase, and *α*-glucosidase in the seminal plasma, indicating a positive impact on the function of the epididymis and accessory glands, particularly the prostate and seminal vesicle, this did not affect semen properties.

Data about the effect of omega-3 and omega-6 PUFAs supplementation on the male reproductive system and semen quality are controversial. In humans, the administration of DHA (1 g/d) and EPA (1 g/d) ameliorated total sperm count, sperm motility, and morphology [46]. Moreover, men supplemented with fish oil exhibited increased testicular size and sperm concentration, along with decreased levels of FSH and LH and elevated free testosterone [47]. Fish oil supplementation has been shown to increase total sperm count, total morphologically normal sperms, and total sperm viability in dogs [48]. However, in boars, fish oil supplementation either reduced the number of morphologically abnormal sperms and improved sperm motility [49] or had no effect on semen quality [50, 51]. In this study, the administration of either omega-3 or sunflower oil for 12 weeks has no obvious effect on sperm count or sperm motility but significantly increased the number of morphologically abnormal sperms indicating spermatogenesis failure.

Kisspeptin is a significant stimulus to the secretion of GnRH and gonadotrophins [52, 53], and a reduction in GnRH and gonadotropin secretions, along with a subsequent decrease in testosterone synthesis by Leydig cells, impairs the process of spermatogenesis. In this study, the administration of omega-3 or sunflower oil inhibited the kisspeptin-GnRH signaling cascade, which further decreases LH, FSH, and testosterone production. Testosterone levels have been shown to increase in rats [54] and decrease in humans [55], dogs [48], and boars [56] after fish oil supplementation. The production of testosterone in Leydig cells is controlled by several genes including *STAR*, *CYP11A1*, *3β-HSD*, *CYP17A1*, and *17β-HSD*. The results herein demonstrated downregulation of these genes by sunflower oil or omega-3 administration, indicating steroidogenesis suppression. Steroidogenesis is reduced by omega-6 PUFAs via its direct effects on StAR and cytochrome P450 [40, 57]. By the nonreversible action of aromatase (CYP19), testosterone can be aromatized to E2 [58]. In this study, the downregulation of testicular *CYP19* mRNA levels contributed to decreased intratesticular levels of E2 following sunflower oil and omega-3 administration. However; the serum levels of E2 were significantly increased, indicating an extratesticular conversion of testosterone to E2. It has been reported that E2 stimulates the secretion of prolactin [59, 60] and increases prolactin mRNA levels [61]. Moreover, acute hyperprolactinemia has been shown to suppress the synthesis of testosterone and male fertility via inhibiting the secretion of GnRH [62], which subsequently inhibits LH pulses [63]. Furthermore, chronic treatment with prolactin has been demonstrated to decrease the expression levels of kisspeptin and GnRH in female mice [64]. Therefore, sunflower oil- and omega-3-induced hyperprolactinemia may be attributed to the repression of kisspeptin-GnRH signaling cascade noticed in this study.

The androgen-androgen receptor (AR) signaling cascade has a fundamental role in the function of male reproductive and nonproductive organs. Activation of the AR mediates testosterone effects, and both androgens and estrogens regulate AR expression in adult rats [65, 66]. Castration has been reported to increase AR mRNA levels [67], and testosterone treatment antagonizes the effect of castration [68]. Estrogen treatment has been shown to increase AR content in the medial amygdala [69] and augment AR mRNA content of the anterior pituitary gland [65]. Furthermore, prolactin induces a dose-dependent increase in nuclear AR content and increases AR mRNA levels in the prostate gland [70, 71]. In the present study, both sunflower oil and omega-3 administration induced a decline in the circulating level of testosterone and an increase in the circulating levels of prolactin and E2. This induction may modulate the expression of ARs in reproductive organs, but 4the intensity of the immunohistochemical expression of ARs in the testes, epididymides, and seminal vesicles (but not prostate) was increased only by omega-3 administration. These results suggest that PUFAs have a tissue-specific effect on ARs that relies on the chemical nature of unsaturated fatty acids. Omega-3 PUFAs have been reported to decrease the number of ARs, as well as plasma testosterone concentrations, in Japanese men [55]. Moreover, omega-3 PUFAs, but not omega-6 PUFAs, are capable of blocking the upregulation of AR gene transcription caused by androgen loss [72].

Inflammation and oxidative stress are known to have adverse impacts on the male reproductive system. The plasma membrane of testicular germ cells consists primarily of PUFAs, which render them susceptible to oxidation by free radicals and thus negatively affect spermatogenesis. Antioxidant enzymes play an integral role in maintaining redox equilibrium. The synergistic interactions of the endogenous enzymatic and nonenzymatic antioxidant systems are measured by TAC [73], and elevated TAC indicates a more efficient antioxidant defense system. In the present study, both sunflower oil and omega-3 administration increased TAC and the activity of the antioxidant defense system, particularly SOD, CAT, and GPx, which prevents the accumulation of H_2_O_2_ and MDA release. A reduction in lipid peroxidation in sperm of dogs [74] and mice [75] has been reported after omega-3 PUFA supplementation. The transcription factor NF-*κ*B functions as a key link between oxidative stress and inflammation [76] and induces various inflammatory genes, such as TNF-*α*, IL-1*β*, and IL-6 [77]. ROS is a key factor for activation of NF-*κ*B [78], which has a negative impact on ROS formation [79], and overexpression of ROS-scavenging enzymes as SOD, GPx, and catalase has been reported to abrogate NF-*κ*B induction [78]; TNF-*α*-mediated production of NF-*κ*B is blunted by CAT [80]. Moreover, H_2_O_2_ has been demonstrated as a second messenger for the signal-induced activation of NF-*κ*B [81] and exposure to oxidants as H_2_O_2_ has been shown to stimulate NF-*κ*B production [82]. In this study, the administration of sunflower oil and omega-3 significantly inhibited NF-*κ*B and downstream cytokine production.

The levels and functional activities of adipokines regulate various signaling systems in the target tissues including the hypothalamic-pituitary-gonadal (HPG) axis. Apelin and its receptor are expressed in the HPG axis, and apelin has been reported to induce infertility via suppressing reproductive hormones including LH, FSH, and testosterone [83, 84]. Moreover, apelin has anti-inflammatory effects [85], stimulates antioxidant enzyme expression, and prevents the production of ROS in adipocytes [86]. Therefore, the increased intratesticular levels of apelin found in this study may have partly contributed to sunflower oil- and omega-3-induced decreases in male reproductive hormones and increases in antioxidant enzyme levels. In particular, apelin has been demonstrated to augment the level of serum adiponectin and reduce leptin [87].

Adiponectin acts as a testicular protective mechanism against the effects of proinflammatory mediators on steroidogenesis [88]. The expression of adiponectin and its receptors in the testis has been reported [22, 89, 90]. Leydig cells are the primary intratesticular source of adiponectin [89]. In the present study, the intratesticular levels of adiponectin were significantly increased following sunflower oil and omega-3 administration. Both basal and GnRH-stimulated LH secretion has been shown to be suppressed by adiponectin [91, 92]. However, adiponectin interacts with kisspeptin-expressing neurons and downregulates kisspeptin1 gene expression, decreasing the stimulatory effect of kisspeptin on GnRH neurons [93]. Therefore, the elevated intratesticular adiponectin levels observed in this study following sunflower oil and omega-3 administration may partially contribute to the inhibition of kisspeptin-GnRH-induced LH production. In rats, a negative relationship between serum adiponectin and testosterone level has been reported [94]. Irisin, an adipo-myokine engaged in energy homeostasis, has various metabolic functions. The injection of exogenous irisin has been shown to boost FSH, LH, and testosterone secretion and ameliorate sperm count and motility in male rats [95]. Furthermore, irisin has shown anti-inflammatory effects via reducing the activity of NF-*κ*B and decreasing the levels of proinflammatory cytokines such as TNF-*α*, IL-1*β*, and IL-6 [96]. In the present study, sunflower oil and omega-3 administration significantly increased the intratesticular levels of irisin, which may play a role in testicular protection via its potential anti-inflammatory effects.

Leptin has been demonstrated to play a direct role in testicular endocrine function and spermatogenesis [97, 98] through its action on the male pituitary-gonadal axis [98]. Leptin stimulates the release of gonadotropins via activation of kisspeptin-induced GnRH neurons in the hypothalamic premammillary nucleus [99]. In the testes, leptin gene expression has been demonstrated in spermatocytes, spermatozoa, and seminiferous tubules [100–102]. In the seminiferous tubules and epididymides, leptin may augment sperm damage via ROS production [103]. In this study, intratesticular leptin concentrations were markedly decreased after sunflower oil and omega-3 administration, suggesting a protective mechanism against ROS generation. Sertoli cell-induced nutritional support of spermatogenesis is amended by leptin [104]. Nevertheless, leptin correlates with reduced sperm motility in seminal plasma [11].

Resistin is a novel adipocytokine with potential implications in metabolic diseases. The expression of resistin in Sertoli and Leydig cells indicates its role in the control of testicular functions [105]. Resistin regulates Leydig cell steroidogenesis and proliferation [106] and increases the synthesis of testosterone [105]. However, seminal resistin concentrations have been shown to negatively correlate with sperm motility and vitality [107]. Resistin has been demonstrated as an inflammation marker, and seminal resistin levels have been shown to positively correlate with the proinflammatory mediators such as elastase, IL-6 [108], and TNF-*α* [107]. In the present study, the administration of either sunflower oil or omega-3 significantly reduced the intratesticular levels of resistin, as well as TNF-*α*, IL-1*β*, IL-6, and NF-*κ*B.

Chemerin is a new adipokine implicated in the regulation of energy metabolism. Chemerin receptors are existent in the testis of rats and humans, and chemerin has been shown to regulate the process of gonadal steroidogenesis in males [12, 109]. In the present study, the administration of sunflower oil or omega-3 significantly reduced the intratesticular concentrations of chemerin. Seminal chemerin concentrations are negatively associated with sperm motility and positively associated with sperm concentration [11].

Visfatin has been recently identified as a novel regulator of the HPG [110] and impacts spermatogenesis [111]. It is present in Leydig cells, spermatocytes, and spermatozoa [12] and has been shown to stimulate steroidogenesis and elevate the production of testosterone in rat cultured Leydig cells [112]. In addition, visfatin mediates inflammatory responses in monocytes via stimulation of proinflammatory and anti-inflammatory cytokines [113]. In this study, the reduced intratesticular levels of visfatin may have partially contributed to the changes observed in proinflammatory cytokines following sunflower oil and omega-3 administration as visfatin has been reported primarily as a proinflammatory marker by increasing the levels of IL-1*β*, IL-6, and TNF-*α* [114, 115].

Vaspin, a member of the serine protease inhibitor family, was found in the epididymal adipose tissue [116]. Human seminal plasma levels of vaspin are positively correlated with sperm DNA fragmentation and negatively correlated with ejaculate volume [11]. Vaspin exerts its anti-inflammatory effects via suppression of TNF-*α*- and IL-1*β*-induced activation of NF-*κ*B [117, 118]. The intratesticular levels of vaspin in this study were repressed after either sunflower oil or omega-3 administration.

## 5. Conclusions

The present study revealed for the first time the potential effects of omega-3 and omega-6 PUFAs on the levels of various adipokines in the male reproductive system. Both omega-3 and omega-6 PUFAs negatively influence the male reproductive function via inhibition of the kisspeptin-GnRH signaling pathway. The consumption of omega-3 and omega-6 PUFAs triggers anti-inflammatory properties via upregulation of apelin, adiponectin, irisin, and IL-10 and downregulation of leptin, resistin, visfatin, prostaglandins, and NF-*κ*B and its downstream elements (IL-1*β*, IL-6, and TNF-*α*). Moreover, both omega-3 and omega-6 PUFAs ameliorate the testicular antioxidant status by scavenging ROS via increasing the levels of enzymes in the endogenous antioxidant system (SOD, CAT, and GPx).

## Figures and Tables

**Table 1:** Specific primers used for the analysis of different gene expressions.

Gene	GenBank accession no.	Primer sequences (5′-3′)	Orientation	Product size (bp)
*STAR*	NM_031558.3	CACAGTCATCACCCATGAGC	Forward	166
		AGCTCTGATGACACCGCTTT	Reverse	
*CYP17A1*	NM_012753	CTCTGGGCACTGCATCAC	Forward	114
		CAAGTAACTCTGCGTGGGT	Reverse	
*CYP19*	NM_017085	GCCTGTCGTGGACTTGGT	Forward	142
		GGTAAATTCATTGGGCTTGG	Reverse	
*3β-HSD*	M38178	TGTGCCAGCCTTCATCTAC	Forward	145
		CTTCTCGGCCATCCTTTT	Reverse	
*17β-HSD*	NM_054007	GACCGCCGATGAGTTTGT	Forward	140
		TTTGGGTGGTGCTGCTGT	Reverse	
*CYP11A1*	J05156	CTTTGGTGCAGGTGGCTAG	Forward	115
		CGGAAGTGCGTGGTGTTT	Reverse	
*GAPDH*	NM_017008.4	GTGCCAGCCTCGTCTCATAG	Forward	122
		CGTTGATGGCAACAATGTCCA	Reverse	

^1^Steroidogenic acute regulatory protein (StAR); 17*α*-hydroxylase/17, 20-lyase (CYP17A1); aromatase cytochrome P450 (CYP19); 3*β*-hydroxysteroid dehydrogenase (3*β*-HSD); 17*β*-hydroxysteroid dehydrogenase (17*β*-HSD); cholesterol side-chain cleavage enzyme (CYP11A1); glyceraldehyde 3-phosphate dehydrogenase (*GAPDH*).

## Data Availability

Data is contained within the article.

## Supplementary Materials

Supplementary Materials Figure S1: graphical abstract showing the effects of omega-3 and omega-6 on testicular levels of adipocytokines, antioxidant status, cytokines, and serum levels of reproductive hormones.
