# Supplementary material for: Effect of Omega-3 or Omega-6 Dietary Supplementation on Testicular Steroidogenesis, Adipokine Network, Cytokines, and Oxidative Stress in Adult Male Rats
Source: Oxid Med Cell Longev. 2021 Jun 28;2021:5570331. doi: 10.1155/2021/5570331 (PMC8260291; doi:10.1155/2021/5570331)
Supplement: Supplementary Materials — Figure S1: graphical abstract showing the effects of omega-3 and omega-6 on testicular levels of adipocytokines, antioxidant status, cytokines, and serum levels of reproductive hormones. [file 5570331.f1.pdf]

Graphical Abstract

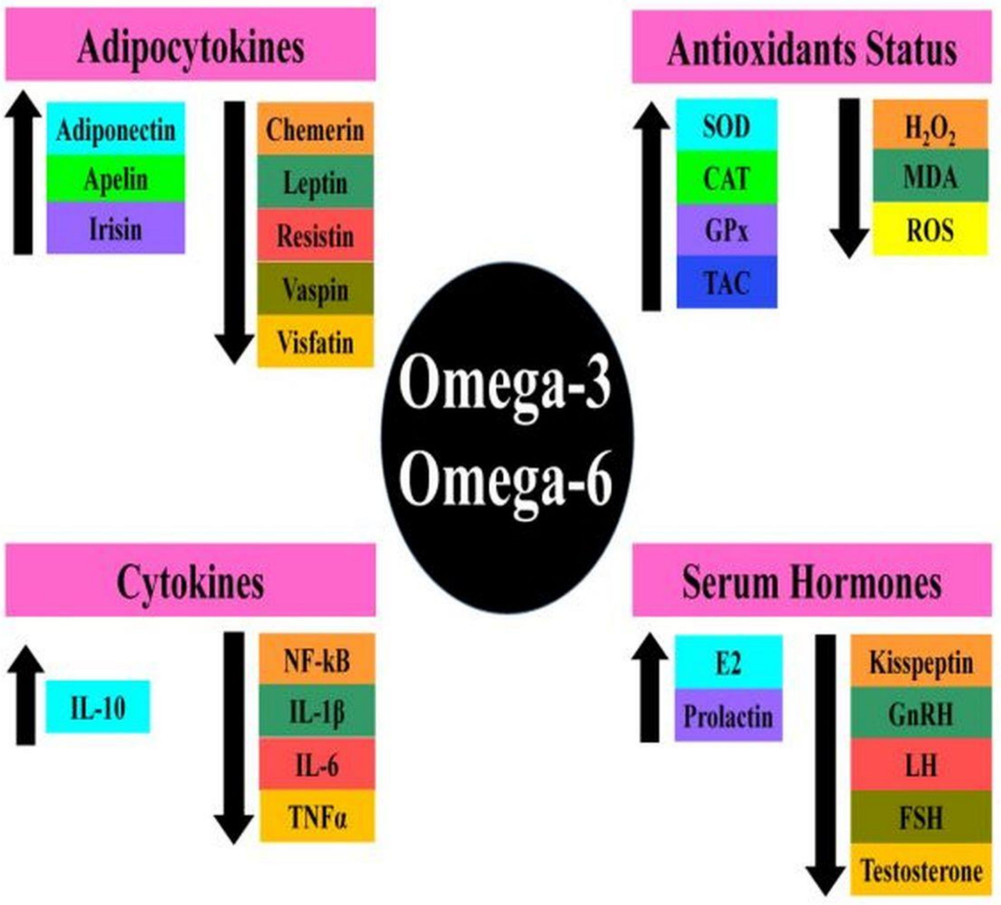


**Supplementary materials**

Graphical abstract showing the effects of omega-3 and omega-6 on testicular levels of adipocytokines, antioxidant status, cytokines, and serum levels of reproductive hormones.
